# The battleship study: An innovation towards precision in prostate biopsy and brachytherapy grid usage

**DOI:** 10.1002/bco2.70279

**Published:** 2026-09-11

**Authors:** Vishen Naidoo, Duvern Ramiah, Marlon Perera, Ahmed Adam

**Affiliations:** ^1^ School of Clinical Medicine, Department of Surgery, Division‐Urology University of the Witwatersrand Johannesburg South Africa; ^2^ School of Clinical Medicine, Department of Radiation Oncology, Academic and Clinical Head of Department University of the Witwatersrand Johannesburg South Africa; ^3^ Division of Oncology, Austin Health, Department of Surgery, Sir Peter MacCallum Cancer Centre, Chair of Prostate Cancer Outcomes University of Melbourne Melbourne Australia; ^4^ Faculty of Clinical Medicine, Department of Surgery, Academic and Clinical Head of Department Division‐Urology, Registry (PCOR‐Vic) University of the Witwatersrand Johannesburg South Africa

**Keywords:** healthcare professional, innovation, low dose rate prostate brachytherapy, MRI fusion template biopsy, novel, operating‐theatre, ProGrid model, prostate brachytherapy, ‘zebra’ grid

## Abstract

**Objectives:**

This study aimed to compare the efficiency and accuracy of a conventional prostate brachytherapy grid with a novel *Zebra* grid using a laboratory‐based simulation model (ProGrid model).

**Methods:**

This prospective and comparative experimental pilot study was conducted using a nonclinical brachytherapy simulation model (ProGrid Model). One hundred medical practitioners (Registrars and Consultants in Urology, Radiation Oncology and General Surgery) were recruited by randomization. Participants performed a series of predetermined coordinate needle insertions using either the conventional grid or the *Zebra* grid. Time taken to identify and accurately insert each coordinate was measured. Demographic and experiential variables were collected via questionnaire. The data were analysed using descriptive statistics, bivariate correlations and multiple regression modelling.

**Results:**

There were no significant differences between groups in baseline characteristics, including level of training, department, brachytherapy experience, gaming experience, hand dominance and use of prescription glasses. The *Zebra* grid demonstrated significantly reduced insertion times across all 10 coordinates compared to the conventional grid (*p* < 0.001). Mean overall time was significantly lower for the *Zebra* grid (3.675 ± 0.9 s) compared to the conventional grid (5.034 ± 1.4 s; *p* < 0.001). No statistically significant differences in accuracy of insertion in the desired grid coordinate were observed between the two grids. Multivariate analysis confirmed grid type as an independent predictor of reduced procedural time.

**Conclusion:**

The *Zebra* brachytherapy grid significantly improves procedural efficiency without compromising accuracy in a simulated setting. This low‐cost, easily reproducible modification has the potential to reduce operating theatre (OT) time and improve workflow in PB. Further clinical studies are recommended to validate these findings and assess their impact on patient outcomes and healthcare resource utilization.

## 1 INTRODUCTION

The global growth in surgical disciplines has revealed equally a vital role of surgery in health care^1^ and the financial struggles in developing and growing surgical facilities.^2^ A large financial outlay and an increased burden to provide the required surgical equipment and trained personnel is incurred in creating more operating time in operating theatres (OT). OTs are a costly element of a hospital due to their specific infrastructure and equipment requirements.^3^ Other factors such as high nurse to patient proportions, the necessity for maintenance and allied services such as transferring patients, linen for theatre as well as environmental hygiene/temperature regulation, cumulatively add to high theatre running costs.

Insight into hospital budgets is a vital component to determine value‐based care. Private hospitals bill insurers and/or patients at a rate higher than expenditure incurred resulting in a profit required to maintain the viability of its business. In the fee‐for‐service model employed in the South African private sector, a higher number of services equate to a greater income.

Operating theatre utilization represents a significant cost driver within healthcare systems. Inefficiencies in theatre result in financial wastage. Such inefficiencies, for example, may refer to time delays before, during or even after the operation. The opportunity cost related to theatre time directly correlates to the forfeiture of income from inefficiency. Minimizing theatre time per case allows the OT to schedule more cases, and hence if efficiency is not maximized, the lost prospective revenue may result in a cost to the hospital. The opportunity costs vary and will be at a maximum for short duration operations.^3^

A study done in 2020 at University of Cape Town, South Africa aimed to assess the cost of OT per minute (regional public hospital). This study model demonstrated a cost of ZAR 31.46/min, and the shorter annualisation model amounted to ZAR 33.77/min^4^ (exchange rate average in 2020, 16.47 ZAR: 1 USD) (South African Reserve Bank).

A comparison in billing from the private sector in South Africa was reviewed as well. The cost from these healthcare providers noted that major theatre time was between ZAR 155/min and ZAR 273/min in 2018.^5^, ^6^, ^7^ The average cost in the private sector was significantly more in comparison with the public sector (exchange rate average in 2019, 14.45 ZAR: 1 USD) (South African Reserve Bank).

Prostate cancer is expected to remain a significant health concern due to an increase in localized disease (low/intermediate risk) being detected requiring active management.^8^

There are various treatment modalities that can be used and numerous factors that are considered in selecting the appropriate patient management, with favourable oncological outcomes. Some factors include the impact that it will have on the patient's quality of life, the treatment time, convenience for the patient and the costs/setup associated with the treatment plan.^9^

The patient will be in a dorsal lithotomy position. A real‐time trans‐rectal probe is inserted into the patient's rectum allowing for the patient's prostate to be visualized in a sagittal and axial plane. A monochromatic brachytherapy applicator template (square grid) is mounted to the trans‐rectal ultrasound probe, which will be positioned in the midline between the scrotum and the anus.

The radioactive sources are inserted into a thin, hollow needle. The coordinates of insertion are mapped out by the medical physicist. The medical physicist and surgeon would have live imaging of the prostate via a trans‐rectal ultrasound probe that is inserted to the patients' rectum. The needles are inserted into the brachytherapy applicator template (square grid) that is positioned on the skin in the area between the anus and scrotum. The radioactive seeds are inserted into the needles via the brachytherapy applicator template (square grid) and into the prostate. The needles are then removed, leaving the radioactive sources in place. They will emit radiation for several months. They are usually considered to be inert after about 6 months. The radiation from the radioactive sources travels a short distance. This in turn allows the seeds/pellets to emit a large amount of radiation within a small space. This restricts the amount of injury to neighbouring healthy tissues. In most cases, there are around 60–80 seeds/pellets that are inserted. However, this is dependent on the size of the prostate and the activity of the sources when the procedure is performed. Because the seeds are so small (4–4.5 mm long and 0.8 mm in diameter), they rarely cause pain. They remain in situ after the prescribed dose of radiation is delivered to the prostate.

The procedure usually takes 1–2 h, and generally, the patient is discharged home the next day. Possible procedural challenges may arise due to the rechecking of accurate grid location as well as precise tracking of the relevant coordinate. The possibility of optimizing a step of the procedure, namely, the insertion of the radioactive source (via the needle), could allow for more patients being done per theatre list. An added benefit would be reduction in theatre time, which would subsequently translate into reduction in theatre cost.

**FIGURE 1:**
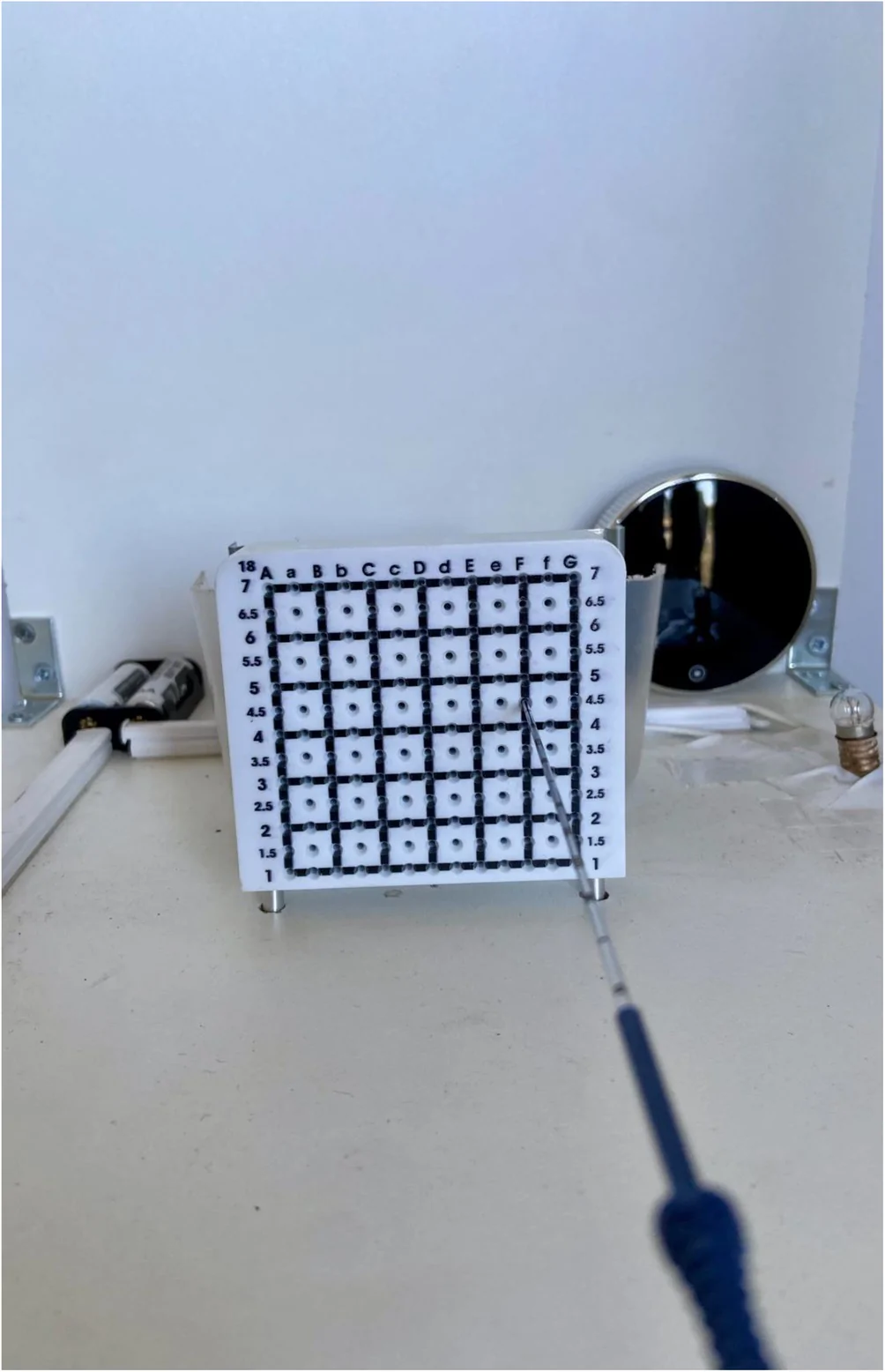
ProGrid model (simulator model).

This study aims to optimize this step by making use of the *Zebra* grid. This is a contrasted (black and white) grid. Higher contrasted objects/images make it easier to discern objects within the image. Black and white provides the maximum contrast and hence will be the basis of the *Zebra* grid.^10^ The *Zebra* grid will be compared to the conventional brachytherapy grid (which is white monochromatic). This comparison will be carried out on a simulator model (ProGrid Model) (see Figure 1) using the relevant equipment, that is, brachytherapy grids and brachytherapy needles. The *Zebra* grid will be prepared by using the same white background grid and 3D printing black lines delineating each row (whole numerical coordinate) and delineating each column (capital alphabetical coordinate). The *Zebra* grid is also reproducible by using a black marker to draw the black lines. The concept of the *Zebra* grid originated during intraoperative discussions between the urology and radiation oncology teams while performing prostate brachytherapy procedures. It was observed that rapid and accurate identification of grid coordinates is a critical step in needle placement and that the monochromatic design of conventional grids may limit visual discrimination under operative conditions. This prompted consideration of whether enhanced visual contrast could improve usability. Input was sought from the Head of Ophthalmology regarding optimal colour contrast for visual clarity. Based on principles of visual perception, black‐and‐white contrast was identified as providing the highest level of discrimination. The *Zebra* grid was subsequently developed using alternating black and white delineations to enhance coordinate visibility, with the aim of improving procedural efficiency without altering the fundamental design of the brachytherapy template.

### 1.1 Study aims

The study compared the conventional prostate brachytherapy grid versus the novel *Zebra* grid. The comparison measured efficiency/time as well as accuracy of insertions for each coordinate given.

### 1.2 Study objectives

1. To conduct an assessment between the two grids
2. To assess accuracy and efficiency (less time taken to complete the *Zebra* grid versus the conventional grid) during the simulations using both grids
3. Administer a questionnaire to participants (variables, which may yield more or less favourable results). (Six qualitative aspect of data collection: level of healthcare provider, department of participant, level of experience doing prostate brachytherapy, previous gaming/console experience, hand dominance and use of prescription glasses)
4. To determine which grid is easier to use and results in less miss ‘hits’

## 2 MATERIALS AND METHODS

### 2.1 Study design

This is a prospective, comparative experimental study. Quantitative and qualitative surveys were completed and analysed. The quantitative aspect is a pilot, lab‐based evaluation of the grids in a non‐clinical setting on simulators (ProGrid Model) and not on patients. The qualitative component is descriptive in nature and limited to participant characteristics collected via a structured questionnaire (see Appendix S1) administered to each participant after assessment of the simulation (control and *Zebra* grid). The data analyzed are quantitative. It makes use of the questionnaire given to each participant after assessment of the simulation (control and *Zebra* grid). Participant selection was voluntary based.

**FIGURE 2:**
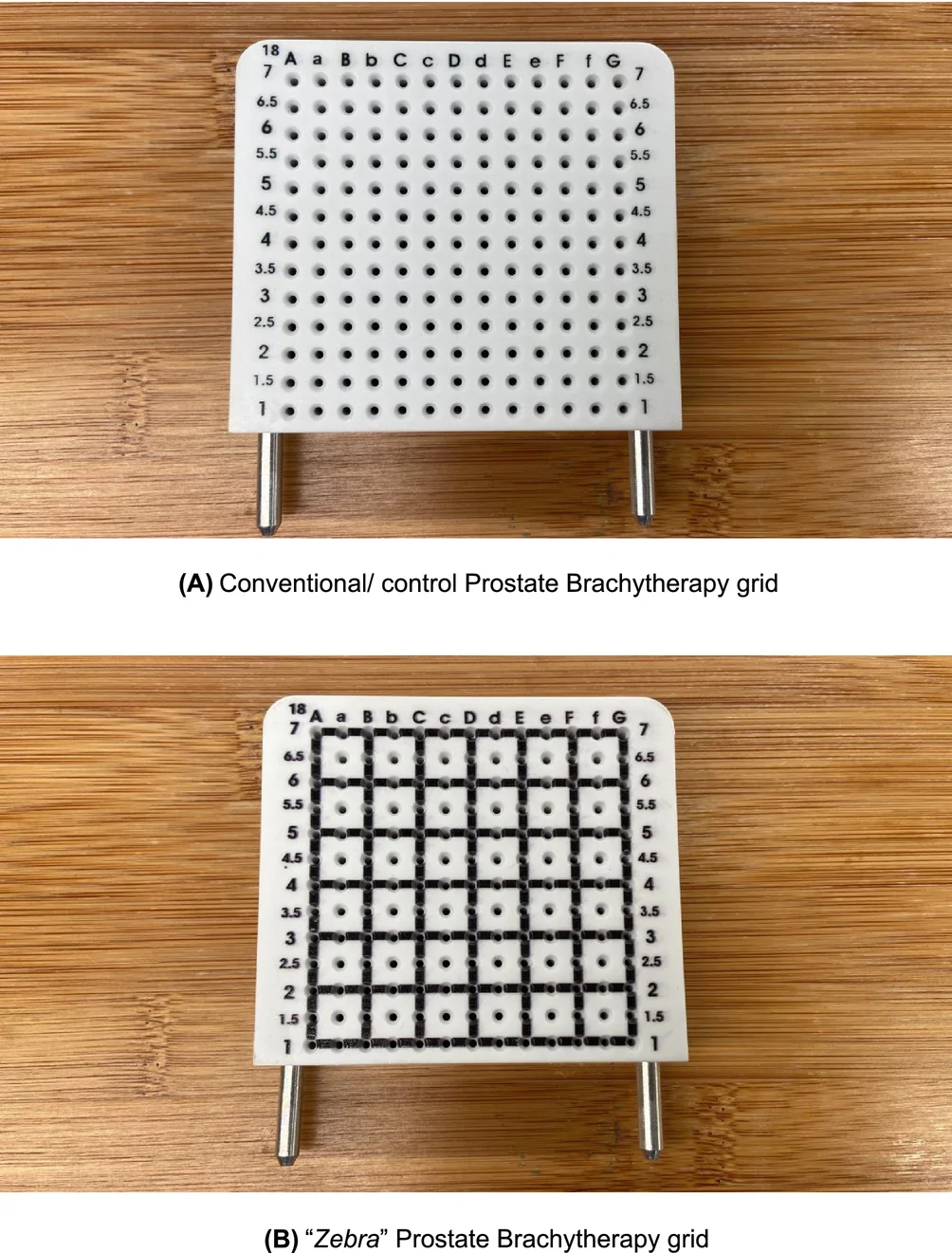
(A) Conventional/control prostate brachytherapy grid. (B) *Zebra* prostate brachytherapy grid.

The study was designed to be a brief, pilot, laboratory‐based simulation with each simulation taking 2–3 min to complete. Each participant was given 10 identical and predetermined coordinates (out of the possible 169 coordinates on a standard brachytherapy grid) for insertion of the brachytherapy needle into the correct coordinate on the grid as determined on the simulation (control and *Zebra* grid). Testing all 169 coordinates would have made each simulation excessively long, which could introduce participant fatigue and limit the feasibility of the study. Accuracy of depth of insertion was not measured as this study's primary endpoint is to evaluate the benefit derived from using a contrasted grid versus a traditional monochromatic grid. The 10 grid coordinates were picked at random and remained the same for each participant. The 10 coordinates used were C4½, E5½, b5½, d3½, A4, F4½, D3, b3, f3 and c5. The comparison measured efficiency/time as well as the accuracy of the insertions for each coordinate given. See Figure 2A,B below, conventional grid and *Zebra* grid.

The first 25 participants (Group 1) performed the control grid, and the second 25 (Group 2) participants performed the *Zebra* grid. The third group of 25 (Group 3) performed the study using either grid, starting with the ‘zebra’ grid and alternating between the two grids, using the same coordinates. The last group of 25 (Group 4) participants performed the study using either grid, starting with the ‘zebra’ grid and alternating between the two grids; however the coordinates followed an inverse order. This aided in eliminating repetition bias and bias that may arise from a more ‘difficult’ prostate mapping (coordinates), which allowed this study to be more robust. The data collected allowed for analysis of accuracy of insertions and if one grid is superior to the other in terms of efficiency.

The mapping of the prostate is a simulated prostate mapping. The same 10 coordinates (in sequence or in an inverse order) were used to simulate one prostate. The entire study conducted used one prostate mapping to avoid bias arising from ‘easier’ or more ‘difficult’ prostate mapping. The simulation model (ProGrid Model) was constructed from two pieces of wood, mounted to each other perpendicularly. The respective grid was secured to the horizontal piece of wood. A metal plate was secured posterior to the grid. The metal plate and brachytherapy needle formed part of the electrical circuit. A voice prompter/recorder read out a new coordinate, in sequence, in 10‐s intervals. The circuit is completed following successful insertion of the needle into the correct coordinate. A light connected to the circuit confirmed accurate insertion. A timer mounted to the ProGrid model measured the time taken to insert the needle into the coordinate. If the participant inserted the needle into the incorrect coordinate, it was tabulated as incorrect, and they proceeded to the next coordinate that was read out after the ten seconds have elapsed. The data were recorded on the data collection sheet (below). The participants answered the questionnaire after the simulation. The study period was completed over 2 months—this allowed for 100 simulations to be performed.

### 2.2 Study population

The target population of this pilot study includes medical healthcare professionals (HCPs) (surgical/urology/radiation oncology registrar and surgical/urology/radiation oncology consultants). Participant selection was random and voluntary based.

### 2.3 Inclusion criteria

1. All participants are medical doctors (Consultant or Registrar in urology, radiation oncology and general surgery).
2. Participants performed the simulation in person at the study site.
3. Participants consented to participate in the study.
4. Participants were able to follow instructions in English and answer the questionnaire in English.

### 2.4 Exclusion from data analysis

1. Incomplete simulations were excluded from the study.
2. Participants who use prescription lenses used their prescription lenses while performing the simulation.
3. Participants were only allowed to perform the simulation once.

### 2.5 Sample size

Sample size calculation using 5% confidence interval.

*Z*‐score—1.96.
E‐margin of error—0.05.
P: standard of deviation—0.5.
N: estimated population (from study population) (surgical/radiation oncology/urology registrars/consultants)

$$\frac{\frac{z^{2}\times P\left(P-1\right)}{E^{2}}\;}{1+\left(\frac{Z^{2}\times P\left(P-1\right)}{E^{2}N}\right)}$$

calculated sample size = 100 participants. Given the pilot nature of the study, a pragmatic sample size of 100 participants was selected to allow for adequate comparison between groups while remaining feasible within the study period.

### 2.6 Data analysis

Both quantitative and qualitative data were collected. Quantitative data (time measured in seconds) were tabulated in Microsoft Excel for 10 grid coordinates. If the participant does not insert the needle into the correct coordinate, it was noted to be incorrect insertion instead of a time point. Mean ± SD for each coordinate was calculated for the control group and the experimental group, along with the mean ± SD of the collective time points of the two groups. Qualitative data gathered from a multiple‐choice questionnaire (Appendix S1) after the experimental process was tabulated in Microsoft Excel. The answers to each MCQ were represented as percentages of the total (control and experimental) sample size. The data are presented as tables to illustrate the differences in demographic of participants more clearly.

## 3 RESULTS

### 3.1 Comparison of qualitative and quantitative data collected

No differences were found between the traditional and *Zebra* grids when comparing level of HCP, department, brachytherapy experience, gaming experience, dominant hand and prescription glasses. This is expected, and statistically confirms the non‐biased, random approach to participant and grid selection.

All time measurements for the 10 coordinates were statistically significantly different, with the time measurements on the *Zebra* grid consistently shorter than those of the conventional grid (see Table 1). The average time measurements for both the conventional (5.034 [1.4]) and *Zebra* (3.675 [0.9]) were also significantly different (*p* < 0.001), with the *Zebra* grid time being shorter than the conventional grid. The observed reduction in mean procedural time highlights the potential clinical significance of the intervention.

**TABLE 1:** Comparisons between the traditional and *zebra* grid.

Variable	Traditional grid (Grid 1)	Zebra grid (Grid 2)	*p*‐Value
Level of HCP (registrar/consultant)	0.098 (0.0)	0.163 (0.0)	0.576
Department (urology/radiation oncology/general surgery)	0.804 (2.0)	1.041 (2.0)	0.232
Brachytherapy experience (weekly/monthly/few times per year/never)	0.255 (0.0)	0.469 (1.0)	0.215
Gaming experience (no/yes)	0.647 (1.0)	0.633 (1.0)	0.904
Dominant hand (left/right/ambidextrous)	0.922 (0.0)	0.939 (0.0)	0.869
Prescription glasses (no/yes)	0.451 (1.0)	0.571 (1.0)	0.301
Coordinate 1 (time) (seconds)	5.384 (2.6)	4.227 (2.4)	0.001
Coordinate 2 (time) (seconds)	4.867 (1.9)	3.800 (1.5)	0.001
Coordinate 3 (time) (seconds)	4.679 (2.4)	3.384 (1.0)	<0.001
Coordinate 4 (time) (seconds)	5.076 (2.2)	3.601 (1.6)	<0.001
Coordinate 5 (time) (seconds)	5.244 (2.5)	3.502 (1.2)	<0.001
Coordinate 6 (time) (seconds)	5.178 (2.0)	3.716 (1.7)	<0.001
Coordinate 7 (time) (seconds)	4.929 (1.9)	3.700 (1.2)	<0.001
Coordinate 8 (time) (seconds)	5.110 (2.3)	3.422 (1.3)	<0.001
Coordinate 9 (time) (seconds)	5.065 (1.9)	4.009 (1.8)	0.001
Coordinate 10 (time) (seconds)	4.950 (1.6)	3.421 (1.6)	<0.001
Average (time) (seconds)	5.034 (1.4)	3.675 (0.9)	<0.001
Coordinate 1 (accuracy) (inaccurate/accurate)	0.961 (0.0)	0.980 (0.0)	0.591
Coordinate 2 (accuracy) (inaccurate/accurate)	1.000 (0.0)	0.980 (0.0)	0.317
Coordinate 3 (accuracy) (inaccurate/accurate)	0.961 (0.0)	1.000 (0.0)	0.168
Coordinate 4 (accuracy) (inaccurate/accurate)	0.961 (0.0)	0.980 (0.0)	0.591
Coordinate 5 (accuracy) (inaccurate/accurate)	0.902 (0.0)	0.980 (0.0)	0.106
Coordinate 6 (accuracy) (inaccurate/accurate)	0.941 (0.0)	0.980 (0.0)	0.335
Coordinate 7 (accuracy) (inaccurate/accurate)	1.000 (0.0)	1.000 (0.0)	1.000
Coordinate 8 (accuracy) (inaccurate/accurate)	0.961 (0.0)	1.000 (0.0)	0.168
Coordinate 9 (accuracy) (inaccurate/accurate)	0.941 (0.0)	1.000 (0.0)	0.088
Coordinate 10 (accuracy) (inaccurate/accurate)	0.961 (0.0)	0.959 (0.0)	0.976

*Note*: Results represented as ‘median (interquartile range = Q3—Q1)’.

Abbreviation: HCP, healthcare professional.

No significant differences in accuracy were observed between the two groups. (Accuracy defined as insertion of the needle tip into the correct coordinate on the first attempt).

### 3.2 Bivariate correlation analyses between all variables studied

#### 3.2.1 Level of HCP

There was no statistically significant correlation between level of HCP (Registrar vs. Consultant) and time taken for any of the 10 coordinates (all *p* > 0.05). Correlation coefficients were weak (R ranging from −0.155 to 0.001), indicating negligible association. (See Table 2.)

**TABLE 2:** Bivariate correlation analyses between all variables studied.

	Valid (*N*)	Spearman (*R*)	*p*‐Value
Level of HCP & Coordinate 1 (time)	97	−0.040	0.697
Level of HCP & Coordinate 2 (time)	99	−0.029	0.773
Level of HCP & Coordinate 3 (time)	98	−0.082	0.420
Level of HCP & Coordinate 4 (time)	97	−0.124	0.227
Level of HCP & Coordinate 5 (time)	95	−0.123	0.235
Level of HCP & Coordinate 6 (time)	95	−0.155	0.133
Level of HCP & Coordinate 7 (time)	100	0.001	0.996
Level of HCP & Coordinate 8 (time)	98	−0.104	0.307
Level of HCP & Coordinate 9 (time)	97	−0.122	0.233
Level of HCP & Coordinate 10 (time)	96	−0.114	0.270
Department & Coordinate 1 (time)	97	−0.291	0.004
Department & Coordinate 2 (time)	99	−0.287	0.004
Department & Coordinate 3 (time)	98	−0.305	0.002
Department & Coordinate 4 (time)	97	−0.169	0.099
Department & Coordinate 5 (time)	95	−0.200	0.052
Department & Coordinate 6 (time)	95	−0.090	0.386
Department & Coordinate 7 (time)	100	−0.106	0.295
Department & Coordinate 8 (time)	98	−0.076	0.460
Department & Coordinate 9 (time)	97	0.041	0.689
Department & Coordinate 10 (time)	96	−0.082	0.429
Brachytherapy experience & Coordinate 1 (time)	97	−0.324	0.001
Brachytherapy experience & Coordinate 2 (time)	99	−0.212	0.035
Brachytherapy experience & Coordinate 3 (time)	98	−0.311	0.002
Brachytherapy experience & Coordinate 4 (time)	97	−0.218	0.032
Brachytherapy experience & Coordinate 5 (time)	95	−0.308	0.002
Brachytherapy experience & Coordinate 6 (time)	95	−0.146	0.158
Brachytherapy experience & Coordinate 7 (time)	100	−0.155	0.125
Brachytherapy experience & Coordinate 8 (time)	98	−0.131	0.198
Brachytherapy experience & Coordinate 9 (time)	97	−0.149	0.144
Brachytherapy experience & Coordinate 10 (time)	96	−0.258	0.011
Gaming experience & Coordinate 1 (time)	97	−0.125	0.221
Gaming experience & Coordinate 2 (time)	99	−0.064	0.529
Gaming experience & Coordinate 3 (time)	98	−0.030	0.768
Gaming experience & Coordinate 4 (time)	97	−0.200	0.050
Gaming experience & Coordinate 5 (time)	95	−0.230	0.025
Gaming experience & Coordinate 6 (time)	95	−0.075	0.469
Gaming experience & Coordinate 7 (time)	100	0.003	0.974
Gaming experience & Coordinate 8 (time)	98	−0.014	0.889
Gaming experience & Coordinate 9 (time)	97	−0.092	0.370
Gaming experience & Coordinate 10 (time)	96	−0.082	0.425
Dominant hand & Coordinate 1 (time)	97	−0.152	0.136
Dominant hand & Coordinate 2 (time)	99	−0.134	0.187
Dominant hand & Coordinate 3 (time)	98	−0.140	0.168
Dominant hand & Coordinate 4 (time)	97	−0.034	0.741
Dominant hand & Coordinate 5 (time)	95	−0.097	0.349
Dominant hand & Coordinate 6 (time)	95	−0.063	0.542
Dominant hand & Coordinate 7 (time)	100	−0.163	0.104
Dominant hand & Coordinate 8 (time)	98	0.072	0.482
Dominant hand & Coordinate 9 (time)	97	−0.007	0.942
Dominant hand & Coordinate 10 (time)	96	−0.074	0.473
Prescription glasses & Coordinate 1 (time)	97	−0.148	0.147
Prescription glasses & Coordinate 2 (time)	99	−0.117	0.250
Prescription glasses & Coordinate 3 (time)	98	−0.208	0.040
Prescription glasses & Coordinate 4 (time)	97	−0.127	0.215
Prescription glasses & Coordinate 5 (time)	95	−0.187	0.070
Prescription glasses & Coordinate 6 (time)	95	−0.108	0.298
Prescription glasses & Coordinate 7 (time)	100	0.003	0.975
Prescription glasses & Coordinate 8 (time)	98	−0.053	0.606
Prescription glasses & Coordinate 9 (time)	97	0.038	0.715
Prescription glasses & Coordinate 10 (time)	96	<0.001	1.000
Grid & Coordinate 1 (time)	97	−0.344	0.001
Grid & Coordinate 2 (time)	99	−0.324	0.001
Grid & Coordinate 3 (time)	98	−0.436	<0.001
Grid & Coordinate 4 (time)	97	−0.499	<0.001
Grid & Coordinate 5 (time)	95	−0.541	<0.001
Grid & Coordinate 6 (time)	95	−0.493	<0.001
Grid & Coordinate 7 (time)	100	−0.421	<0.001
Grid & Coordinate 8 (time)	98	−0.502	<0.001
Grid & Coordinate 9 (time)	97	−0.351	<0.001
Grid & Coordinate 10 (time)	96	−0.474	<0.001

Abbreviation: HCP, healthcare professional.

Interpretation: Level of training did not influence procedural efficiency, suggesting that both junior and senior clinicians performed similarly in the simulated environment.

#### 3.2.2 Department

A statistically significant negative correlation was observed between department and time taken for Coordinates 1, 2 and 3 (*R* ≈ −0.29 to −0.31; *p* ≤ 0.004). No significant correlations were found for the remaining coordinates. (See Table 2.)

Interpretation: Participants from certain departments (those with more procedural exposure, such as Urology or Radiation Oncology) demonstrated slightly faster performance in early tasks. However, this effect was not sustained across all coordinates, suggesting a limited or learning‐phase influence rather than a consistent determinant of performance.

#### 3.2.3 Brachytherapy experience

Brachytherapy experience showed statistically significant negative correlations with time for Coordinates 1, 2, 3, 4, 5 and 10 (*R* ≈ −0.21 to −0.32; *p* < 0.05). No significant correlations were seen for Coordinates 6–9. (See Table 2.)

Interpretation: Greater experience in brachytherapy was associated with faster performance, particularly in earlier coordinates. This likely reflects improved familiarity with grid navigation and procedural workflow. The diminishing significance in later coordinates may indicate a learning or adaptation effect among less experienced participants.

#### 3.2.4 Gaming experience

Gaming experience demonstrated limited influence on performance. Statistically significant correlations were only observed for Coordinates 4 (*p* = 0.050) and 5 (*R* = −0.230; *p* = 0.025), with weak negative correlations. All other coordinates showed no significant association. (See Table 2.)

Interpretation: While gaming experience may confer minor advantages in hand–eye coordination or spatial awareness, its overall impact on procedural efficiency was minimal and inconsistent.

#### 3.2.5 Dominant hand

No statistically significant correlations were observed between dominant hand and time taken for any coordinate (all *p* > 0.05), with weak correlation coefficients. (See Table 2.)

Interpretation: Hand dominance did not influence procedural performance, suggesting that the task design did not favour either left‐ or right‐handed participants.

#### 3.2.6 Prescription glasses

A statistically significant correlation was observed only for Coordinate 3 (*R* = −0.208; *p* = 0.040), with no significant associations for the remaining coordinates. (See Table 2.)

Interpretation: Visual acuity (as inferred from use of prescription glasses) did not meaningfully impact performance overall. The isolated finding is likely incidental and not clinically significant.

#### 3.2.7 Grid type

Grid type demonstrated the strongest and most consistent correlations with procedural time across all coordinates. Statistically significant moderate negative correlations were observed for all 10 coordinates (R ranging from −0.324 to −0.541; all *p* ≤ 0.001). (See Table 2.)

Interpretation: The negative correlation indicates that use of the *Zebra* grid was consistently associated with reduced time to complete each coordinate. This represents the most important finding in the analysis and confirms that grid design is a key determinant of procedural efficiency.

### 3.3 Overall interpretation

These findings seen in Table 2 suggest that modification of equipment design (i.e., the *Zebra* grid) has a greater impact on procedural efficiency than operator‐related factors, supporting the hypothesis that improved visual contrast enhances task performance.

## 4 MULTIPLE REGRESSION MODEL ANALYSIS

Multiple regression analysis demonstrated that grid type was the only variable consistently associated with reduced procedural time across all coordinates and in the overall average model (all *p* ≤ 0.034). Use of the *Zebra* grid was independently associated with significantly shorter completion times. Brachytherapy experience showed a significant association with average time (*p* = 0.025) and at Coordinate 10 (*p* = 0.022) but was not consistently significant across all coordinates. Overall, grid design emerged as the strongest independent predictor of procedural efficiency. (See Table 3.)

**TABLE 3:** Multiple regression model for average (time).

*N* = 100	*β*	Standard error of *β*	*b*	Standard error of *b*	*p*‐Value
Department	0.033	0.096	0.043	0.126	0.732
Brachytherapy experience	−0.222	0.098	−0.439	0.193	0.025
Gaming experience	−0.108	0.084	−0.270	0.209	0.201
Prescription glasses	−0.026	0.085	−0.063	0.204	0.758
Grid	−0.530	0.083	−1.272	0.200	<0.001

## 5 DISCUSSION

This study demonstrates a strong and statistically significant improvement in procedural efficiency when using the novel *Zebra* prostate brachytherapy grid compared to the conventional monochromatic grid, without compromising accuracy. These findings are particularly relevant in the context of prostate brachytherapy, where procedural precision and efficiency are crucial to ensure optimal oncological outcomes and minimize treatment‐related morbidity.

The primary finding of this study is the consistent reduction in time required to accurately identify and access grid coordinates when using the *Zebra* grid. Across all 10 coordinates, as well as in the overall average time, the *Zebra* grid significantly outperformed the conventional grid (*p* < 0.001). This implies that enhanced visual contrast facilitates improved spatial recognition and hand–eye coordination during needle placement. High‐contrast visual environments have been shown to improve task performance and object discrimination, particularly in precision‐based tasks. The alternating black‐and‐white pattern likely enhances coordinate delineation, reducing the cognitive effort required for grid navigation. In high‐volume clinical settings, even small reductions in time per coordinate may cumulatively translate into meaningful reductions in total procedural time.

Importantly, this improvement in efficiency did not come at the cost of accuracy. There was no statistically significant difference in correct versus incorrect insertions between the two grids. This finding is clinically significant, as accuracy in prostate brachytherapy is critical for achieving appropriate dosimetric coverage while minimizing radiation exposure to surrounding structures such as the rectum and bladder. The absence of a difference in accuracy may also reflect the controlled simulation environment and lack of considerable time pressure, suggesting that both grids are inherently reliable, with differences primarily related to usability rather than precision.

The regression and correlation analysis further support the robustness of these findings. Grid type remained an independent predictor of reduced procedural time across multiple coordinates and in the overall model, even after adjusting for confounding variables such as the participant's previous brachytherapy experience and demographic factors. This strengthens the conclusion that the observed improvements in efficiency are attributable to the design characteristics of the *Zebra* grid rather than participant‐related variability.

As expected, increased brachytherapy experience was associated with shorter completion times, highlighting the importance of procedural familiarity and skill acquisition, demonstrating that operator experience is an important determinant of efficiency in surgical and procedural settings. However, even after accounting for experience, the *Zebra* grid retained a statistically significant independent effect on performance. Additionally, factors such as dominant hand, use of prescription glasses, level of training and gaming experience did not significantly influence outcomes, suggesting that the benefit of the *Zebra* grid is applicable across different user groups.

From a clinical and health systems perspective, these findings have important implications. Operating theatre time represents a major cost driver in healthcare systems. Previous studies have demonstrated that operating theatre costs may range from approximately ZAR 31–34/min in the public sector to between ZAR 155 and ZAR 273/min in the private sector.^6^, ^7^, ^8^, ^9^ Inefficiencies in theatre utilization contribute to financial waste and reduced patient access to surgical care.^3^, ^5^ Therefore, improvements in procedural efficiency, even if modest on an individual level, may result in substantial cost savings and improved resource utilization when applied across multiple cases. It is unlikely to increase same day surgical volume, and while the mean time savings of 1.3 s per insertion may result in ~500 ZAR saving per procedure. This may not be the driver of major revenue but serves as proof that the efficiency gain incurs no added operational cost penalty, yielding a net‐positive workflow benefit over high annual volumes. Furthermore, while efficiencies are minor in isolation, any reduction in total anaesthesia time and patient positioning in lithotomy position contributes incrementally to patient safety and recovery.

Enhanced visual cues can improve task efficiency and reduce cognitive load, particularly in environments requiring rapid and precise decision‐making. The *Zebra* grid represents a simple, low‐cost innovation that leverages this principle. Unlike more complex technological advancements, such as robotic systems or advanced imaging platforms, this modification is easily reproducible and does not require additional infrastructure or specialized training. This makes it particularly suitable for implementation in both high‐resource and resource‐limited healthcare settings.

Despite these strengths, several limitations should be considered. Firstly, this study was conducted in a simulated, non‐clinical environment. While simulation allows for standardization and control of variables, it does not fully replicate the complexities of live prostate brachytherapy, including patient movement, anatomical variability and real‐time imaging challenges. As such, the observed efficiency gains may differ in clinical practice.

Secondly, the study design required removal of the needle after each coordinate insertion, which differs from standard clinical practice where needles are typically left in situ during seed placement. Depth of insertion differs in clinical practice for each needle and seed insertion, and this was not simulated in this study. Efficiency was measured solely in terms of 2D coordinate targeting (positioning the needle tip at the specified entry trajectory) rather than true seed placement efficiency. The simulation did not account for insertion depth or seed release mechanics; hence the reported time savings reflect spatial needle positioning rather than the complete clinical delivery process. Future iterations incorporating depth‐controlled insertion will better capture the full procedural time dynamics.

Thirdly, although the sample size was adequate for a pilot study, larger studies would be beneficial to confirm these findings and explore potential subgroup differences in greater detail. Furthermore, the inclusion of qualitative user feedback regarding ease of use, confidence and preference could provide additional insight into the practical advantages of the *Zebra* grid.

Future research should focus on evaluating the *Zebra* grid in a clinical setting. A prospective comparative study assessing operative time, dosimetric outcomes, complication rates and user satisfaction would be valuable in determining its clinical utility.

## 6 CONCLUSION

This study demonstrates that the novel *Zebra* prostate brachytherapy grid considerably improves procedural efficiency compared to the conventional grid, without compromising accuracy. The enhanced visual contrast of the *Zebra* grid facilitates faster coordinate identification and needle placement, resulting in consistently reduced completion times across all measured parameters.

The findings suggest that this simple, low‐cost modification has the potential to improve workflow efficiency in prostate brachytherapy. In clinical practice, this may translate into reduced operating theatre time, improved patient throughput and potential cost savings, particularly in resource‐constrained healthcare environments.

Importantly, the benefits of the *Zebra* grid are independent of operator experience, making it a universally applicable tool across various levels of training and specialties. This enhances its potential for widespread adoption. Despite freehand perineal prostate biopsy becoming the standard of care, the trans‐perineal grid template biopsy may also be beneficial if extensive prostate mapping is required for patients on active surveillance/small peripheral lesions or patients with repeat negative biopsies.

While the results are promising, they are based on a simulation model and should be interpreted within this context. Further clinical studies are required to validate these findings in real‐world settings and to assess their impact on patient outcomes and procedural logistics.

In conclusion, the *Zebra* grid represents an effective and practical innovation in prostate brachytherapy. Its implementation may offer a meaningful improvement in procedural efficiency while maintaining the high standards of accuracy required for safe and effective cancer treatment.

## AUTHOR CONTRIBUTIONS

Ahmed Adam and Duvern Ramiah conceived the idea as well as the study design. Vishen Naidoo collected the data, analysis and write up of the manuscript. Ahmed Adam, Duvern Ramiah and Marlon Perera reviewed and assisted with preparation of the manuscript.

## CONFLICT OF INTEREST STATEMENT

The authors declare no conflicts of interest.

## DISCLOSURE

Naidoo declare that this research report is my own work. This work has not been submitted before for any degree or examination at any other University or Journal. Naidoo further declare that all sources used or quoted have been indicated and acknowledged by means of complete references. Naidoo understand that plagiarism is wrong and that this work may be checked electronically for originality.

## Supporting information


**Appendix S1.** Supporting Information

## Data Availability

The data that support the findings of this study are available from the corresponding author upon reasonable request.
