# Supplementary material for: The battleship study: An innovation towards precision in prostate biopsy and brachytherapy grid usage
Source: BJUI Compass. 2026 Sep 11;7(9):e70279. doi: 10.1002/bco2.70279 (PMC13564107; doi:10.1002/bco2.70279)
Supplement: Supplementary file 1 [file BCO2-7-e70279-s001.docx]

**Appendices-**

Appendix A- User evaluation questionnaire + Data collection sheets

**Appendix A**

**Questionnaire/ data collection sheet**

**Level of health care provider**

[consultant] [registrar]

**Department**

[urology] [radiation oncology] [General Surgery]

| No. | Question |  |
| --- | --- | --- |
| Q.1 | level of experience doing brachytherapy | [weekly] [monthly] [few times per year] [never] |
| Q.2 | Previous gaming/console experience | [yes] [no] |
| Q.3 | Which is your dominant hand? | [right] [left] [ambidextrous] |
| Q.4 | Do you wear prescription glasses | [yes] [no] |

**Data collection sheet 1**

| **Control GRID** | Accuracy [yes] [no] | Time [seconds] |
| --- | --- | --- |
| Coordinate 1 |  |  |
| Coordinate 2 |  |  |
| Coordinate 3 |  |  |
| Coordinate 4 |  |  |
| Coordinate 5 |  |  |
| Coordinate 6 |  |  |
| Coordinate 7 |  |  |
| Coordinate 8 |  |  |
| Coordinate 9 |  |  |
| Coordinate 10 |  |  |

**Data collection sheet 2**

| **Zebra (contrasted) Grid** | Accuracy [yes] [no] | Time [seconds] |
| --- | --- | --- |
| Coordinate 1 |  |  |
| Coordinate 2 |  |  |
| Coordinate 3 |  |  |
| Coordinate 4 |  |  |
| Coordinate 5 |  |  |
| Coordinate 6 |  |  |
| Coordinate 7 |  |  |
| Coordinate 8 |  |  |
| Coordinate 9 |  |  |
| Coordinate 10 |  |  |
